# Differential contributions of dorsolateral and frontopolar cortices to working memory processes in the primate

**DOI:** 10.3389/fnsys.2015.00144

**Published:** 2015-10-29

**Authors:** Erica A. Boschin, Mark J. Buckley

**Affiliations:** Department of Experimental Psychology, University of OxfordOxford, UK

**Keywords:** prefrontal cortex, frontopolar cortex, dorsolateral prefrontal, delay, valuation

## Abstract

The ability to maintain and manipulate information across temporal delays is a fundamental requirement to bridge the gap between perception and action. In the case of higher-order behavior, the maintenance of rules and strategies is particularly helpful in bridging this gap. The prefrontal cortex (PFC) has long been considered critical for such processes, and research has focused on different subdivisions of PFC to gain an insight into their diverse contributions to these mechanisms. Substantial evidence indicates that dorsolateral PFC (dlPFC) is an important structure for maintaining information across delays, with cells actively firing across delays and lesions to this region causing deficits in tasks involving delayed responses and maintenance of rules online. Frontopolar cortex (FP), on the other hand, appears to show the opposite pattern of results, with cells not firing across delays and lesions to this region not affecting the same rule-based, delayed response tasks that are impaired following dlPFC lesions. The body of evidence therefore suggests that dlPFC and FP’s contributions to working memory differ. In this article, we will provide a perspective on how these regions might implement distinct but complementary and interactive functions that contribute to more general temporally-extended processes and support flexible, dynamic behavior.

## Working Memory and Prefrontal Cortex (PFC)

A fundamental aspect of cognition is the ability to maintain and manipulate information even when it cannot be directly perceived in the form of sensory input, for example because it is no longer accessible. Besides contributing to basic memory processes, such as the passive maintenance of information for future use, this type of cognitive processing is also essential in order to associate actions and/or stimuli with outcomes that may be temporally distant from the onset of the action or stimulus themselves. Furthermore, it is advantageous for the planning and execution of sequential behavioral plans that span longer timescales than that of a single action.

The prefrontal cortex (PFC) has long been considered critical for this cognitive ability, often referred to by the very general and umbrella term “working memory”. Several studies have linked PFC cells’ activities with the internal representation of information, ranging from the encoding of stimulus features, to value, to more abstract rules, goals and strategies (Asaad et al., 1998, 2000; White and Wise, 1999; Wallis et al., 2001; Bunge et al., 2003; Kennerley et al., 2011), as well as with the maintenance and manipulation of information across time (Fuster and Alexander, 1971; Goldman-Rakic, 1995; Miller et al., 1996; Bunge et al., 2003; Mushiake et al., 2006; Mansouri et al., 2007). PFC damage in human patients has been linked to severe deficits in memory and planning (Bauer and Fuster, 1976; Goldman-Rakic, 2011; Fuster, 2008; Thompson-Schill et al., 2002) and such patterns of impairment have also been extensively reported in the animal literature (for a comprehensive review, see Fuster, 2008). In particular, the effects of large targeted PFC ablations on a range of tasks in non-human primates have led some authors to hypothesize a role for PFC in processing specifically temporally extended and/or temporally complex information (Wilson et al., 2010).

## Dorsolateral and Frontopolar Cortices and Temporally Extended Prefrontal Functions

**Figure 1:**
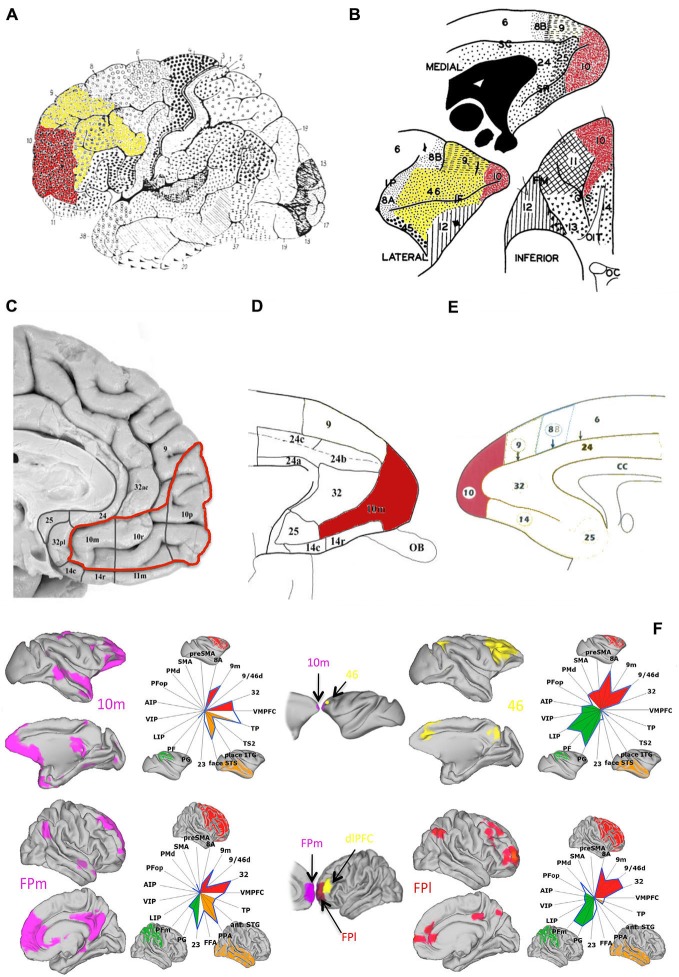
**Anatomy and connectivity of prefrontal cortex (PFC) in the human and monkey brain. (A)** Lateral view of human brain (adapted from Brodmann (1909), pp. 108, Figure 85, with permission from Springer): frontopolar cortex (FP) (red) is visible at the most anterior portion of the frontal lobe, identified approximately as Brodmann area 10, with dlPFC (yellow) occupying the area immediately posterior and superior to FP. **(B)** Lateral, medial and inferior view of the macaque’s PFC (adapted from Walker (1940), with permission from Wiley): FP (red) is visible at the tip of the macaque’s frontal lobe and dlPFC (yellow) is visible in the tissue above and surrounding the principal sulcus. **(C–E)** Medial view of the human **(C)** (adapted from Ongür et al. (2003), with permission from Wiley) and macaque **(D,E)** (adapted from Ongür et al. (2003) and Petrides and Pandya (1999), respectively, with permission from Wiley) PFC: FP (red) extends rostrally into the medial surface of the PFC according to some cytoarchitectonical subdivisions (**C,D**—areas 10r and 10m). **(F)** Mapping of resting-state functional connectivity of FP (medial—left, in purple—and lateral—right, in red) with more posterior areas, comparing connectivity in the macaque brain (top) with the human brain (bottom). Spider plots illustrate the intensities of the coupling patterns between FP (location of the seed regions are illustrated in the central column, following the same color scheme) and the target regions of interest. The connectivity profile of human medial FP (FPm) closely resembles that of medial area 10 (10m) the macaque brain. Human lateral FP (FPl), on the other hand, appears to resemble macaque area 46, here shown in yellow (adapted from Neubert et al., 2014, with permission from Elsevier).

Evidence suggests that, rather than being a functionally homogeneous region, PFC may comprise a network of cytoarchitecturally and functionally distinct subdivisions (Walker, 1940; Carmichael and Price, 1994; Petrides and Pandya, 2002; Petrides, 2005; Brodmann, 1909). Therefore, one question concerns whether particular subdivisions of PFC might be specifically crucial for particular processes referred to under the general rubric of working memory processes. Fuster, 2008 distinguished between lateral prefrontal and medial prefrontal syndromes, with the former, but not the latter, being characterized by impairments in, amongst other functions, working memory. Indeed, a large number of findings regarding the properties of PFC cells and the effects of PFC damage on working memory tasks come from investigations into lateral PFC, and particularly the dorsolateral prefrontal (dlPFC) regions (Figures 1A–E) including, in the macaque, the area surrounding the principal sulcus (Petrides, 2000). Human neuroimaging studies have shown that a region anteriorly adjacent to dlPFC, namely frontopolar cortex (FP), approximately corresponding to Brodmann’s area 10 (Figures 1A–E), is also particularly active during working memory and episodic memory tasks in humans (Gilbert et al., 2006a,b) and it has been associated with prospective memory (PM) functions, i.e., the maintenance of information related to a future action plan across time-delays (Okuda et al., 2007; Burgess et al., 2011; Volle et al., 2011). Consistent with Fuster’s distinction between lateral and medial PFC syndromes, FP’s memory functions have also generally been associated with its lateral portion, which, in humans, has been found to closely resemble macaque’s dorsolateral area 46 in terms of functional connectivity with wider cerebral cortex (Figure 1F; Sallet et al., 2013; Neubert et al., 2014).

Nevertheless, recent studies have also begun to highlight some differences between the two regions, for example in neurophysiological profiles of cells in dlPFC vs. FP. Cells in the dorsal and lateral aspect of FP, unlike more posterior cells in dlPFC *per se*, do not appear to fire across temporal delays (Tsujimoto et al., 2010, 2012), which is a property generally deemed characteristic of temporally extended memory processes. It is therefore possible that dlPFC and FP might be supporting different processes contributing to more general memory functions. One way to investigate this possibility is to look at the effects of selective lesions to each of these two areas on the performance of the same type of cognitive tasks, in order to discern whether their respective contributions can be differentiated. While several experiments have investigated the effects of dlPFC lesions on various components of working memory, up until very recently, the absence of studies on the effects of targeted FP lesions had precluded such a comparison. In the light of new experimental findings, we can now begin to form some hypotheses on the potential distinct contributions of these two regions to cognition.

## Stimulus Features

In tasks of recognition memory such as delay-matching-to-sample (DMS) or delay-non-matching-to-sample (DNMS), the subject has to maintain a memory trace of the perceptual features of a sample stimulus, in order to accurately compare them with those of a test stimulus (or stimuli) after delays of varying length. Cells in dlPFC have been shown to fire during delays in such tasks, with activity correlated to the individual properties of the sample (Miller et al., 1996; Sawaguchi and Yamane, 1999). In a series of classic studies, Fuster and colleagues showed that, in the monkey, cooling of dlPFC regions including sulcal area 46 caused deficits in spatial delayed-response and DMS tasks with increased delays, but not on simultaneous matching-to-sample tasks (Fuster and Alexander, 1970; Bauer and Fuster, 1976). Further investigations have suggested a more nuanced role for dlPFC in DMS/DNMS tasks than that of passive general maintenance of information, as lesions to dlPFC can leave performance on these tasks relatively unimpaired (Passingham, 1975; Bachevalier and Mishkin, 1986; Kowalska et al., 1991), but can affect specific processes that contribute to DMS/DNMS performance, such as visuospatial processes (Passingham, 1975; Levy and Goldman-Rakic, 2000) or the selection and manipulation of information that is maintained “online” across temporal delays in order to guide choice behavior (Petrides, 2000; Rowe et al., 2000).

**Figure 2:**
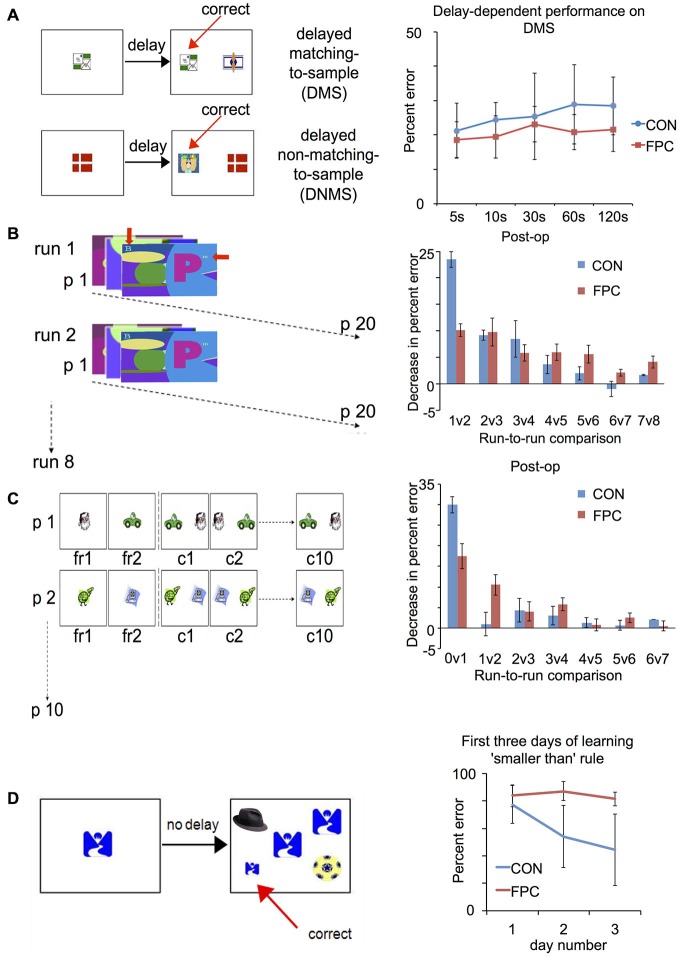
**Patterns of spared and impaired performance following FP lesion in the macaque (adapted from Boschin et al., 2015): tasks (right) and results (left). (A)** Delayed-Matching/Delayed-Non-Matching-to-Sample: FP animals are not impaired compared to controls across several different delays. **(B)** Objects-in-scenes: in this task, animals learn about which of a pair of foreground objects (alphanumeric characters, indicated by the red arrows) presented within a complex scene is associated with reward. They are presented with 20 novel problems every day and in each daily session they are tested on that set of problems eight times. Animals are tested for 15 days pre-operatively and post-operatively. For control animals, the greatest improvement in performance (measured as decrease in percent error) was observed between the first and second run, indicating rapid learning. FP animals, on the other hand, did not show such substantial improvement between the first and second run, indicating a deficit in rapidly learning about the relative values of novel stimuli. **(C)** Successive single-problem learning. The animals learn about which of a single pair objects (clipart images) is associated with reward with problems presented successively. In the first run they are given forced-choice trials where the rewarded and unrewarded item are presented individually (order counter-balanced across trials), then they are tested on that problem 10 times successively. A session comprises 10 such problems and each animal completes 10 sessions pre- and post-operatively. FP animals were again impaired on rapid, one-trial learning about the relative value of novel stimuli, (here measured as the decrease in percent error between the forced-choice phase and the first presentation of a problem between the two stimuli). **(D)** Acquisition of a new abstract rule: animals are trained to perform a simultaneous matching-to-sample task requiring them to choose a stimulus on the basis of two concurrent abstract rules (“matching” and “smaller than”). As an intermediate phase they are trained on the new “smaller-than” rule for 3 days, which is depicted in this figure. Control animals showed a significant decrease in percent error from the first to the second day of learning to apply the new “smaller than” rule. This is indicative of rapid learning about the value of the novel abstract rule. FP animals, however, did not display such an improvement.

While no recordings of FP cells during DMS/DNMS task exist to date, we recently investigated the effects of targeted lesions to the macaque’s FP on both tasks, and found that, unlike dlPFC lesions, these had no effect on any aspect of the animals’ performance of either task (Figure 2A). The FP animals were undistinguishable from controls both in reaching criterion for the tasks and in their performance across varying delays (Boschin et al., 2015). This suggests that, despite its activation during working memory tasks, FP is not essential to support the maintenance of visual information across delays, nor for guiding choice behavior based on the type of visual information and rules that underpin DMS/DNMS tasks.

## Abstract Rules and Strategies

The need to maintain or manipulate information across time is not exclusively a requirement of situations where one needs to hold a memory trace of a cue or stimulus that can no longer be directly perceived, as in the case of DMS/DNMS tasks. Even in the presence of constant sensory input, in the form of visual stimuli for example, other types of task-relevant information might be maintained, such as rules, strategies or action plans. A large body of evidence does implicate both dlPFC and FP in the encoding, maintenance and manipulation of task instructions, abstract rules and strategies (Rowe et al., 2000; Strange et al., 2001; Wallis et al., 2001; Mushiake et al., 2006; Sakai and Passingham, 2006; Christoff and Keramatian, 2007; Rowe et al., 2007; Sakai, 2007; Buckley et al., 2009; Tsujimoto et al., 2011; Mian et al., 2012), and one hypothesis about FP function posits that this area sits atop of a prefrontal hierarchy where increasingly abstract information is represented in rostral vs. caudal PFC regions (Badre and D’Esposito, 2007; Koechlin and Summerfield, 2007; Badre, 2008). Therefore one possibility is that FP’s role in temporally extended cognitive processing can only be uncovered when the task involves a higher level of abstraction than in DMS/DNMS.

While any type of rule-based behavior benefits from reliable and consistent maintenance of rules and context across time, this type of processing is particularly useful in situations where rules or instructions are not explicitly cued on every trial and/or are not kept constant, but, rather, change dynamically. While in versions of DMS/DNMS when the rule varies from trial-to-trial, but is nonetheless cued, significant BOLD activity is elicited in ventral PFC but not dlPFC (Bunge et al., 2003), activity in dlPFC is found in contexts where rules are not explicitly cued and, for example, have to be inferred by stay/switch cues (Forstmann et al., 2005), have to be learnt by trial-an-error (Monchi et al., 2001; Lie et al., 2006), or have to be decided for oneself (Bengtsson et al., 2009). Furthermore, FP cells have been shown to increase activity when feedback indicates that responses are correct according to the current strategy, but only when they are not directly cued (Tsujimoto et al., 2010, 2012). Therefore, both dlPFC and FP appear to be more engaged in contexts where uncued behavioral alternatives have to be maintained and differentially selected depending on changes in contextual demands.

Variants of the Wisconsin Card Sorting Test (WCST)—where subjects are required to respond by matching a sample to one of several test items according to uncued rules that vary dynamically across the session—have proved valuable in animal and human neuropsychological studies investigating the underlying neural mechanisms supporting such behavior. In a monkey-analog of the WCST, single-cell recordings in the macaque’s principal sulcus (area 46 and 9/46) have identified cells that encode and maintain a representation of the currently relevant rule both within and between trials (Mansouri et al., 2006) and, in a conflict-version of the task, a representation of the level of conflict experienced on the current and previous trials was also found in the same area (Mansouri et al., 2007). Consistent with these findings, lesions to this region impair the animal’s ability to maintain the rule in memory across increasing delays (Buckley et al., 2009), as well as the ability to adapt behavior in response to varying levels of conflict (Mansouri et al., 2007). This indicates that the monkey principal sulcus is essential for supporting the maintenance and exploitation of dynamically changing task rules and task-relevant contextual information across time.

As in the case of DMS/DNMS tasks, to date no recordings have been carried out in the macaque FP during the WCST analog. However, recent findings about the effects of lesions to this area indicate that, unlike dlPFC lesions, FP damage does not impair animals on either rule maintenance or rule switching in the standard version, nor does it impair the conflict version of the task (Mansouri et al., 2015). This may be seen as further consistent with findings reporting neurons that encode rules and strategies in dlPFC but not in FP (Mansouri et al., 2006; Tsujimoto et al., 2010, 2011, 2012).

Nevertheless, while FP animals were not *impaired* in any aspect of the WCST analogs, FP lesions did nonetheless have an effect on performance, in the form of an *enhancement* compared to controls. FP lesioned animals were better at adapting their behavior following exposure to conflict and were also less susceptible to intervening distractors, regardless of salience, being better able to maintain the relevant rule in memory compared to controls (Mansouri et al., 2015). This pattern of enhancements after FP lesions, contrasted with the pattern of impairments following dlPFC lesions in the same task, suggests that, while dlPFC seems to be fundamental for maintaining and selecting the appropriate behavioral strategies, FP may play a very different role in this type of abstract and dynamic cognitive behavior.

## Evaluating the Relative Value of Novel Alternatives: a Proposed Contribution of Frontopolar Cortex to Cognition

We hypothesize that a key contribution of FP to cognition is in supporting the exploration and evaluation of the relative value of different alternatives, particularly when novel. This hypothesis is supported by the effects of FP lesions across a range of behavioral tasks, in particular the findings of very specific effects of such lesions on rapid learning about novel alternatives across three different tasks: an objects-in-scenes task (Figure 2B), a successive single-problem learning task (Figure 2C), and the acquisition of a new abstract rule (“smaller than”) in a simultaneous visual discrimination task (Figure 2D; Boschin et al., 2015).

In these tasks, control animals showed a sharp decrease in errors in the early stages of choosing between new alternative scenes and objects, or acquiring a novel alternative rule, indicating that they were able to rapidly extract information about the relative value of these novel alternatives. FP lesioned animals, on the other hand, showed no such pattern of rapid learning (see Figures 2B–D), but were indistinguishable from controls in later stages of learning, where error rates decreased more gradually (Boschin et al., 2015). This indicates that FP might be crucial for a mechanism that aids the rapid extraction of the relative value of different behavioral options, above and beyond the kind that can be implemented through repeated, direct experience with the outcome of each alternative. This mechanism might involve the computation of internal inferences about the value of unchosen alternatives relative to the value of those that have been directly chosen. Animals with an intact FP might be at an advantage compared to animals without an FP because they are able to infer more about the potential value of unchosen options based on their experience with the chosen option.

This hypothesis is consistent with the data from Mansouri et al. (2015) about the enhancing effects of FP lesions in contexts where distractors (such as free reward and novel tasks between trials of the WCST) may represent alternatives that the animal perceives as being potentially relevant to goal-directed behavior. If, as we hypothesize, FP is involved in the ongoing process of evaluating alternatives in relation to one another, it would be expected to both facilitate rapid learning about novel alternatives, as well as bias animals to explore the potential value of novel alternatives that turn out to be mere distractors. Therefore, animals without a FP would not be biased in such a manner and better able to exploit reward opportunities from ongoing goal-directed behavior when faced with distraction, as demonstrated by Mansouri et al. (2015). Similarly, they could better adapt their behavior to varying levels of conflict, in the absence of the deleterious effects of distraction (Mansouri et al., 2015). Indeed, patients with lesions to FP have been found to perform better than controls in tasks that involve concentration (Petrie, 1952; Burgess et al., 2012). This would also be consistent with Rowe et al. (2007) findings that patients with FP lesions made fewer errors than controls on “stay” trials, but more errors on “switch” trials, which is consistent with the idea of increased focus on the current task set ignoring potential alternatives. Indeed, FP activity in human subjects was recently found to be correlated with the difference in value between chosen vs. unchosen options (Boorman et al., 2009, 2011) as well as with exploratory behavior (Daw et al., 2006) and changes in FP functional connectivity were reported when subjects switch to a previously unchosen alternative (Boorman et al., 2009).

This new framework could allow for new interpretation of some influential findings regarding the activation of FP in tasks with a working memory component. For example, Volle et al. (2011) showed that patients with FP lesions were impaired on a PM task where they were asked to perform stimulus-judgments while concurrently maintaining the intention to push a button every 30 s. Importantly, they were not impaired when the PM task was explicitly cued by a visual stimulus (i.e., pressing a button whenever they saw an animal). Our hypothesis of FP function could help explain these findings in a novel way as, in the time-based PM task, patients would have had to continually maintain and assess the relative value of the two tasks (stimulus-judgement vs. button-press), which fluctuated depending on the recency of the latest button-press, whereas no such requirement was present in the event-based PM task, where the value of the prospective memory task was explicit when cued.

## Conclusions and Future Direction

Taken together, the evidence we presented can be interpreted within a theoretical framework where FP and dlPFC support distinct, but complementary and interactive, cognitive processes that can contribute to more general temporally extended functions, namely the exploration and evaluation of the value of novel behavioral alternatives and the implementation of ongoing behavior based upon what is perceived to be the contextually most relevant information, respectively. In tasks where action plans can span long timeframes and/or need to be updated dynamically in response to contextual changes, dlPFC is essential to appropriately maintain, select and manipulate information, rules and behavioral strategies, particularly in the absence of specific cues that inform the subject about the most appropriate response. In these dynamic contexts, FP can interact with dlPFC by providing the latter with information about novel valuable behavioral options that dlPFC can then encode, maintain and implement in order to flexibly adapt behavior.

Regarding generalization across species, comparative functional connectivity studies have suggested that while human medial FP resembles macaque FP, human lateral FP resembles dorsolateral area 46 in the macaque as opposed to macaque FP (Neubert et al., 2014). However, our findings (Boschin et al., 2015) are consistent with the human imaging literature about lateral and medial FP function (Boorman et al., 2009, 2011). Further, the effects of FP lesions doubly-dissociate from the effects of lesions to posteriorly adjacent dorsolateral areas in the macaque (i.e., FP lesions impair rapid scene learning but not short-term rule-memory, whereas principal sulcus lesions show the reverse pattern of impairments; see Baxter et al., 2008; Buckley et al., 2009; Boschin et al., 2015; Mansouri et al., 2015), consistent with existing literature regarding dlPFC’s role in the maintenance, manipulation and selection of information, rules and strategies (e.g., Rowe et al., 2000; Petrides, 2000; Forstmann et al., 2005; Bengtsson et al., 2009). Therefore, from a functional point of view, there appears to be consistency across species about the role of these two areas in behavior. One possibility is that the differences in connectivity observed in Neubert et al.’s (2014) study were confounded by differences in the cognitive states of the subjects (i.e., anesthetized animals vs. restive awake humans). This question certainly deserves further investigation and an important part of future research will be to directly relate findings from human and animal studies in the same brain-state, ideally an active state associated with ongoing choice-behavior.

Moving forward in the exploration of the role of dlPFC and FP in these processes, the key concept is *interaction*. Most of the data collected so far has stemmed from the study of individual areas in isolation, but neuroimaging in humans has begun to draw attention to the highly interactive nature of activity between PFC and wider cortical networks (Sakai and Passingham, 2006; Rowe et al., 2007; Boorman et al., 2011). For example, Sakai and Passingham (2006) showed that FP appears to influence posterior regions differently depending on the intended rule to be implemented via context-dependent changes in functional connectivity between FP and different task-relevant posterior regions. Furthermore, Rowe et al. (2007) showed in a related paradigm that when FP was damaged regions posterior to FP also interacted with each other differently. However, such data remains correlative. New experimental methodologies now offer scope to investigate how different regions causally influence the areas to which they are connected (and vice-versa) when animals engage in choice behavior, by employing a combination of simultaneous multi-neuronal recordings and reversible inactivations and/or lesions during the same behavioral tasks. Besides their functional differences, FP and dlPFC also present differences in their anatomical connections with other regions. In terms of the specific areas they are connected to, dlPFC’s connections span a wide network of both cortical and subcortical structures (Masterman and Cummings, 1997; Petrides and Pandya, 1999; Yeterian et al., 2012), while FP’s connections are more robust with higher-order prefrontal regions and are considerably sparser in more posterior and subcortical regions (Petrides and Pandya, 2007; Burman et al., 2011a,b; Yeterian et al., 2012). Furthermore, even for regions that are connected to both FP and dlPFC, there can be differences at the level of synaptic connectivity Medalla and Barbas (2010). Therefore, combining selective inactivation of FP and dlPFC with recordings, should help shed light not only on their individual functions, but on how the neural dynamics in the areas interconnected with these regions are differentially affected when the former is inactivated as opposed to the latter, and how that might also affect the way the interconnected region of interest interacts with its own different target areas. For neuroscience to progress, we strongly support the notion that a paradigm shift is required away from investigating individual regions in isolation towards investigating how areas interact at the neuronal level both in the healthy brain and in the face of brain damage, dysfunction and disease.

## Conflict of Interest Statement

The authors declare that the research was conducted in the absence of any commercial or financial relationships that could be construed as a potential conflict of interest.
